# Wilms Tumor with Vena Caval Intravascular Extension: A Surgical Perspective

**DOI:** 10.3390/children11080896

**Published:** 2024-07-25

**Authors:** Daniel B. Gehle, Zachary D. Morrison, Huma F. Halepota, Akshita Kumar, Clark Gwaltney, Matthew J. Krasin, Dylan E. Graetz, Teresa Santiago, Umar S. Boston, Andrew M. Davidoff, Andrew J. Murphy

**Affiliations:** 1 Department of Surgery, St. Jude Children’s Research Hospital, Memphis, TN 38105, USA; daniel.gehle@stjude.org (D.B.G.); zachary.morrison@stjude.org (Z.D.M.); huma.halepota@stjude.org (H.F.H.); clark.gwaltney@stjude.org (C.G.); andrew.davidoff@stjude.org (A.M.D.); 2 Department of Surgery, University of Tennessee Health Science Center, Memphis, TN 38163, USA; 3 Department of Surgery, Division of Pediatric Surgery, University of Tennessee Health Science Center, Memphis, TN 38163, USA; jadekumar@uthsc.edu; 4 Department of Radiation Oncology, St. Jude Children’s Research Hospital, Memphis, TN 38105, USA; matthew.krasin@stjude.org; 5 Department of Oncology, Solid Tumor Division, St. Jude Children’s Research Hospital, Memphis, TN 38105, USA; dylan.graetz@stjude.org; 6 Department of Pathology, St. Jude Children’s Research Hospital, Memphis, TN 38105, USA; teresa.santiago@stjude.org; 7 Department of Surgery, Division of Pediatric Cardiothoracic Surgery, University of Tennessee Health Science Center, Memphis, TN 38163, USA; uboston@uthsc.edu

**Keywords:** Wilms tumor, nephroblastoma, inferior vena cava, vascular extension, pediatric cancer, pediatric surgery

## Abstract

Wilms tumor (WT) is the most common kidney tumor in pediatric patients. Intravascular extension of WT above the level of the renal veins is a rare manifestation that complicates surgical management. Patients with intravascular extension are frequently asymptomatic at diagnosis, and tumor thrombus extension is usually diagnosed by imaging. Neoadjuvant chemotherapy is indicated for thrombus extension above the level of the hepatic veins and often leads to thrombus regression, obviating the need for cardiopulmonary bypass in cases of cardiac thrombus at diagnosis. In cases of tumor extension to the retrohepatic cava, neoadjuvant therapy is not strictly indicated, but it may facilitate the regression of tumor thrombi, making resection safer. Hepatic vascular isolation and cardiopulmonary bypass increase the risk of bleeding and other complications when utilized for tumor thrombectomy. Fortunately, WT patients with vena caval with or with intracardiac extension have similar overall and event-free survival when compared to patients with WT without intravascular extension when thrombectomy is successfully performed. Still, patients with metastatic disease at presentation or unfavorable histology suffer relatively poor outcomes. Dedicated pediatric surgical oncology and pediatric cardiothoracic surgery teams, in conjunction with multimodal therapy directed by a multidisciplinary team, are preferred for optimized outcomes in this patient population.

## 1. Introduction

Wilms tumor (WT), also known as nephroblastoma, is the second-most common extracranial pediatric solid tumor behind neuroblastoma. WT comprises approximately 5–6% of all childhood malignancies before age 15 years, with most cases presenting before 6 years of age [1,2,3]. Intravascular tumor thrombus extension into the renal veins and the inferior vena cava (IVC), sometimes progressing to the right atrium (RA), is a rare, advanced presentation of Wilms tumor that carries significant potential for surgical morbidity and presents a unique challenge to pediatric surgeons. Contemporary practice typically consists of neoadjuvant chemotherapy prior to oncologic resection [4]. This approach frequently results in thrombus regression; however, in cases of persistent tumor thrombus extension to the supradiaphragmatic IVC and/or RA, sternotomy and cardiopulmonary bypass (CPB) and possibly circulatory arrest will be required. Despite being a locally advanced disease with the potential for increased surgical morbidity, intravascular extension beyond the renal veins does not seem to portend particularly increased overall mortality when thrombectomy is successfully performed, according to many studies. In this review, we provide a brief background on WT with intravascular tumor extension and detail the unique diagnostic and technical surgical considerations of this disease entity. 

## 2. Epidemiology

Wilms tumor with malignant IVC thrombus occurs in 4–10% of all patients diagnosed with WT, with cephalad cardiac extension rarer at 1–3% of cases [5,6,7,8,9,10,11]. In one of the largest retrospective cohort studies of patients from the National Wilms Tumor Study 4 (NWTS-4), Shamberger et al. identified that 165 of 2731 patients (6%) were found to have intravascular extension to the IVC (134 patients, 4.9%) or RA (31 patients, 1.1%) [8]. RA extension has been reported to have higher prevalences in smaller single-center cohorts outside of North America and Europe, ranging from 2.5 to 19%, which may be due to delayed presentation in low- and middle-income countries [11,12,13,14,15,16,17]. Patients with WT with intravascular extension tend to present at a median age of 4 to 5 years, slightly higher than the overall ages of presentation for WT [5,6,7,15,17,18,19,20,21]. Though older age is a poor prognostic factor in patients with WT, intravascular invasion in and of itself is not when multidisciplinary care and resources are available [22]. The male–female ratio for patients with intravascular extension is roughly even, and though a slightly higher prevalence in females has been found in many studies, no meta-analysis of this potential trend has been performed to our knowledge [7,9,15,20]. There appears to be a propensity for tumor thrombus in right-sided tumors, perhaps due to the shorter renal vein [6,15,18,19]. Bilateral Wilms tumor (BWT) does not appear to predispose to intravascular extension; patients with BWT from the NWTS-3 study had a rate of IVC thrombus of 10/152 (6.6%), which is similar to the overall rates [23,24]. Other cohorts of patients with suprarenal intravascular tumor extension have identified frequencies of BWT ranging from 3 to 45% (with most <15%), and these are similar to the overall proportions of BWT [4,5,7,25,26,27].

## 3. Presentation and Diagnosis

Most patients with WT and cavoatrial tumor thrombus are diagnosed with preoperative imaging studies and do not exhibit signs or symptoms specific to caval occlusion or invasion. Clinical signs suggestive of cardiac involvement may be more common when extensive intravascular involvement occurs, presenting as hemodynamic instability or cardiac failure; IVC syndrome with lower extremity swelling and visible abdominal wall collateral veins; or manifestations of Budd–Chiari syndrome such as jaundice, ascites, abdominal pain, and/or hepatic encephalopathy [28]. 

In a retrospective cohort study by Cristofani et al. of 16 patients with IVC (8) or RA (8) involvement, 2/16 (12.5%) had heart failure as a presenting symptom [15]. Nakayama and associates reviewed the records of 15 patients from the first three North American-based NWTS studies with cardiac extension [10]. Five of these fifteen patients had ascites at presentation, four had pleural effusions, three exhibited hepatomegaly, and one patient had hypotension with a murmur. In a later review of patients from the NWTS-3 study, most patients presented with an abdominal mass (42%) or gross hematuria (30%). Few patients presented with signs more specific to intravascular tumor involvement: 2/77 (3%) with varicocele, 2/77 (3%) with hepatomegaly secondary to hepatic vein obstruction, and 1/77 (1%) with congestive heart failure from a RA thrombus [24]. Another retrospective study of patients from the NWTS-4 study noted that only 6/165 (4%) patients with confirmed intravascular extension exhibited suggestive signs preoperatively including ascites, dilated venous collaterals, or varicocele [8]. In a more recently published multicenter study of 69 patients with IVC and/or cardiac thrombus, 14% presented with signs of IVC thrombosis such as Budd–Chiari syndrome, evidence of venous collaterals, or lower extremity edema [20]. Other studies have corroborated this relatively low incidence of specific symptoms with cavoatrial thrombus [6,15,28,29,30,31].

The usual diagnostic evaluation for children who present with an abdominal mass begins with abdominal ultrasound (US) with color Doppler sonography, followed by IV contrast-enhanced abdominopelvic computed tomography (CT) or magnetic resonance imaging (MRI) and chest CT [6,32,33,34]. Identification of a contiguous, enhancing mass within an enlarged renal vein or IVC on contrasted CT is pathognomonic for tumor thrombus; however, compression of vascular structures from mass effect due to a large primary tumor and/or bulky regional lymphadenopathy may limit evaluation in some cases [35,36,37]. Other radiographic features seen with cavoatrial involvement include azygos vein enlargement, retroperitoneal collateral venous circulation, or unilateral renal hypoenhancement due to venous outflow obstruction. Echocardiography is valuable and sensitive in the further evaluation of patients with suspected intracardiac extension of tumor thrombus [24].

The accuracy of imaging modalities for the diagnosis of tumor thrombus and intravascular extension is variable when compared to the gold standard reference of intravascular extension identified intraoperatively and on the final pathology. An early review of 77 patients from the NWTS-3 study with intravascular extension identified sensitivities of 59% (33/56) for US, 42% (16/38) for CT, 87% (20/23) for venacavography, 56% (5/9) for angiography, and 100% (2/2) for MRI [24]. A 2015 study of 82 patients from the North American-based AREN03B2 study comparing MRI and CT modalities for locoregional staging found that both MRI and CT correctly identified the one patient confirmed to have tumor thrombus extension to the IVC but did not identify the three patients with tumor thrombus at the level of the renal vein [37]. In a larger study of patients from AREN03B2 published in 2012, Khanna and colleagues evaluated the accuracy of CT and renal Doppler US in a total of 173 patients with WT (62 with intravascular extension at time of surgery) who underwent nephrectomy and identified a sensitivity/specificity of 65.6%/84.8% for CT and 45.8%/95.7% for Doppler US for the detection of tumor thrombus (renal vein or cavoatrial involvement) in the primary nephrectomy group [38]. Sensitivities were slightly higher when limited to the cavoatrial subgroup, with 11/13 identified by CT and 7/10 via Doppler. However, in this study, for patients who underwent delayed nephrectomy due to the identification of tumor thrombus above the level of the hepatic veins, initial unresectability, or other indications, the sensitivity/specificity of tumor thrombus was higher for both modalities with 86.7%/90.6% for CT and 66.7%/100.0% for Doppler US [38]. Finally, in cases where tumor thrombus was identified preoperatively by CT, accuracy for the most distal extent of thrombus was reasonably high at 81% (17/21) in the primary surgery group and 89% (23/26) in the secondary surgery group. 

Improvement in the sensitivity and specificity of CT modality in the study by Khanna et al. when compared to the 1988 study by Ritchey et al. can perhaps be explained by advances in CT imaging protocols and technology. Of note, overall, the detection of intravascular tumor extension is higher than individual modality sensitivities, as most patients undergo multiple preoperative imaging studies. When there is diagnostic uncertainty from other modalities regarding the cephalad extent of tumor thrombus, venacavography has been utilized. However, this method’s interpretability is limited when there is significant caval local compression or when the child cries if the study is not performed under general anesthesia [39,40]. This invasive modality’s utilization is likely to continue to decrease with continued advancements and experience with US, CT, and MRI. 

Transesophageal echocardiography (TEE) is particularly advantageous, as it can be performed intraoperatively. Disadvantages of TEE include requiring at least moderate anesthesia, its limited utility with retrohepatic or lower thrombus, and its advanced training requirements compared to transthoracic echocardiography. TEE use has been better described in adult oncology and pediatric congenital heart disease populations, and its sensitivity and specificity have not been evaluated in patients with WT and intravascular extension [41,42,43].

## 4. Classification and Staging

The staging criteria for WT with intravascular extension are based on the distal extent of tumor thrombus and the completeness of resection. A major paradigm difference between the management of WT in North America and Europe is the preferential utilization of preoperative chemotherapy in Europe according to International Society of Paediatric Oncology (SIOP) guidelines, whereas neoadjuvant chemotherapy is reserved for particular cases according to North American Children’s Oncology Group (COG) standards [44]. These organizations have staging systems that are similar for WT with intravascular extension, but the COG system is based on pre-chemotherapy evaluation, while SIOP is based on post-chemotherapy surgical evaluation [45,46]. Both of these systems assign a local and an overall disease stage, with the local stage defined by the extent of abdominal disease and the overall stage incorporating the local stage, the presence of distant metastases, and/or bilateral disease [47,48]. Patients with tumor thrombus involving the renal vein, IVC, or with intracardiac extension who undergo complete en bloc (not piecemeal) resection with negative gross and microscopic margins are classified as local stage II disease in both systems. It is important to consider that, after the renal vein or IVC is cut, the vessel’s wall retracts, and the thrombus usually protrudes beyond the margin in the final specimen. Yet, if the thrombus is not transected and the vascular margin is negative, this is still designated as local stage II. According to both standards, stage III disease includes those who undergo preoperative biopsy, positive microscopic or gross margins upon resection, or those with incompletely resected disease due to invasion into vital structures. Similarly, both systems classify stage III disease as regional lymph node involvement confined to the abdomen, tumor spillage intraoperatively, or peritoneal implants. In contrast, stage IV disease is diagnosed with distant lymphatic or hematogenous metastases outside the abdominal cavity.

Tumor histology is an important factor for prognostication and adjuvant chemotherapy management in patients with WT, and there may be an association of tumor histology subtype with the presence of intravascular extension. The COG and SIOP paradigms for classifying tumor histology are similar but exhibit some key differences. The COG classification is based on the absence (favorable histology) or presence (unfavorable histology) of anaplastic histologic components in the tumor. The SIOP classification includes eight histological subtypes based on neoadjuvant chemotherapy-induced changes and viable tumor components, which are then grouped into low-, intermediate-, and high-risk types. Both groups utilize histology classification with other clinicopathologic factors (such as tumor volume, stage, and metastatic foci chemotherapy response in both systems with the addition of molecular markers and patient age according to COG) to determine adjuvant therapy for individual patients [49]. Shamberger et al. found that NWTS-4 patients with intravascular extension had slightly higher rates of favorable histology (92.1%, 140/152) and slightly lower rates of diffuse anaplasia (4.6%, 7/152) when compared to those without intravascular extension (87.5%, 1372/1568 and 7.5%, 118/1568, respectively), although significance testing was not completed [8]. In another large study, Meier et al. found significant differences in histologic subtypes among patients with intermediate-risk WT with or without vena caval thrombus wherein patients with caval thrombus had higher proportions of regressive or completely necrotic histology and lower proportions of mixed-type histology in the final pathology reports [21]. This study and others have, importantly, also found significantly increased rates of metastatic disease to the liver and lungs and a larger primary tumor volume in patients with caval thrombus, which may be at least partially explained by the later presentation or older age of these patients at diagnosis [5,15,21,25].

**Figure 1:**
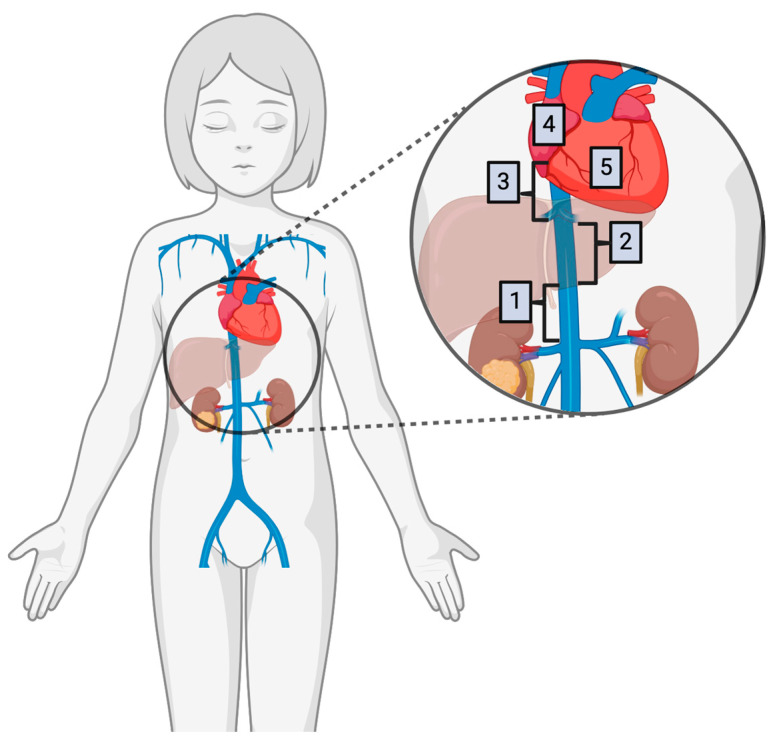
Diagram of Wilms tumor intravascular involvement classification system proposed by Abdullah et al. [11] wherein (1) indicates infrahepatic, (2) indicates retrohepatic, (3) indicates suprahepatic, (4) indicates right atrial, and (5) indicates right ventricular tumor thrombus extension. Created with BioRender.com.

The degree of intravascular tumor extension has been further classified to facilitate surgical planning. Some studies have utilized a classification system originally described by Hinman, with level 1 being intrahepatic thrombus, level 2 being suprahepatic and subdiaphragmatic, and level 3 being supradiaphragmatic involvement [28,50]. Daum et al. in 1994 proposed four “stages” (separate from the COG and SIOP stages, as above), including tumor thrombus up to the level of subhepatic IVC (1), retrohepatic IVC (2), suprahepatic IVC (3), or the level of the heart (4) [51]. In the discussion of a later case series published by Abdullah et al. in 2013, the authors proposed addition of a Stage 5 to further stratify intracardiac extension into right atrial involvement (4) and right ventricular involvement (5) due to changes in operative approach and anesthetic concerns due to tricuspid valve obstruction with positive pressure ventilation [11]. This classification scheme is represented in Figure 1. These authors also proposed sub-stage classification denoted as A, B, or C, representing the absence (A) or presence (B) of tumor thrombus adherence/infiltration to the vessel wall or hepatic vein involvement (C) [11]. 

## 5. Management

### 5.1. Role of Preoperative Chemotherapy

Neoadjuvant chemotherapy is utilized in nearly all cases of WT in Europe according to SIOP guidelines and protocols, with few exceptions based on patient age and radiographic criteria. In North America, neoadjuvant chemotherapy is explicitly recommended according to COG guidelines in cases of WT with intravascular involvement when tumor thrombus is present at the level of the hepatic veins or more cephalad, to potentially reduce the extent of tumor thrombus. Tumor thrombus extension above the level of the hepatic veins complicates thrombectomy by requiring intrapericardial caval control or cardiopulmonary bypass. However, contemporary series from the United States also demonstrate that neoadjuvant chemotherapy is commonly utilized for retrohepatic or infrahepatic vena caval tumor thrombus involvement [4]. Regression of the tumor thrombus may reduce surgical complexity by eliminating the need for sternotomy, cardiopulmonary bypass (CPB), and/or vascular reconstruction [8,24,39]. Evidence suggests that standard courses of chemotherapy lasting 4–6 weeks have comparable outcomes to extended chemotherapy courses lasting > 6 weeks with regards to tumor thrombus regression and completeness of resection, with more favorable event-free survival (EFS) and overall survival (OS) with a standard number of courses [52]. According to a recent systematic review and meta-analysis published in 2021 by Boam et al., neoadjuvant chemotherapy also reduces the rates of viable tumor thrombus on the final pathology by approximately one-half, with extended courses of chemotherapy trending towards increased tumor thrombus viability when compared to the standard courses (odds ratio 3.14, 95% CI 0.97 to 10.16, *p* = 0.056) [53]. Neoadjuvant therapy has also been shown to reduce the size of the primary tumor, which confers benefits of easier mobilization of the kidney and tumor and less distortion of normal anatomy intraoperatively [25].

Importantly, the decision to administer neoadjuvant chemotherapy or proceed with upfront resection is nuanced. In certain cases of symptomatic disease, the clinical presentation may warrant foregoing neoadjuvant therapy and proceeding with urgent primary resection due to the risk of distal embolism from an unstable tumor thrombus, traumatic or spontaneous tumor rupture, or acute illness from IVC or hepatic vein thrombosis, such as in cases of Budd–Chiari syndrome [11,25,29,54,55]. Conversely, Shamberger et al. identified a complication rate of 25/96 (26.0%) in children undergoing upfront resection of WT with associated intravascular tumor thrombus and in 9/68 (13.2%) receiving neoadjuvant therapy that trended towards significance. This risk of upfront surgical resection was mitigated when complications from neoadjuvant chemotherapy were included, for a total complication rate of 13/69 (18.8%) in the neoadjuvant therapy group [8]. Deaths from neoadjuvant chemotherapy toxicity and complications unrelated to tumor thrombus have been reported in other cohorts [17,27]. Other large multicenter studies have not adequately reported or captured complications related to neoadjuvant chemotherapy, so further conclusions regarding the risks of neoadjuvant chemotherapy in this population are limited [4].

Overall, tumor thrombus partial or complete response occurs at high rates in response to neoadjuvant chemotherapy, with a significant proportion of cases of intracardiac extension at diagnosis avoiding the eventual need for CPB. Rates of complete resolution of tumor thrombus have been reported at 0% to 40% [4,5,15,17,26,27], and multiple cohorts have demonstrated that complete thrombus resolution occurs most frequently in patients with infrahepatic IVC thrombus at diagnosis [4,5,26]. Rates of either partial (PR) or complete thrombus response (CR) vary widely, with combined rates ranging from 18 to 90%. A study by Qureshi et al. saw higher PR or CR rates in cardiac level thrombi than in the infrahepatic group, although no significance testing was done [30]. A recent large multicenter study by Pio et al. found similar rates of PR or CR between IVC and cardiac thrombus groups [20]. Such variability in responses to chemotherapy between centers and studies can likely be attributed to the underlying biological heterogeneity of WT, relatively small number of patients in single-center series, variability between centers in neoadjuvant chemotherapy regimens, and imaging modalities. Key large and multicenter studies assessing the tumor thrombus response to neoadjuvant therapy are highlighted in Table 1. Our search strategy for the tabulation of relevant papers included in Table 1 was as follows: PubMed and SCOPUS were queried with the search terms “Wilms tumor” or “nephroblastoma” in combination with “intracaval”, “caval”, “intracardiac”, “cardiac”, “cavoatrial”, “IVC”, “intravenous”, or “intravascular” with or without “involvement” or “extension” in various combinations. We reviewed citations and abstracts from this search strategy, and bibliographies were screened for additional relevant literature. Case reports, reviews, and case series with five or fewer patients were omitted from further review. Studies were reviewed if published after 1990 and if abstracts included outcomes following the administration of neoadjuvant chemotherapy.

### 5.2. Surgical Approaches to Thrombectomy

WT resection in cases with intravascular extension is associated with higher rates of perioperative complications than in cases without intravascular extension, but comparable long-term outcomes can be achieved with en bloc complete resection of the primary tumor and thrombus in conjunction with multimodal therapy. The surgical approach depends on the level of intravascular tumor thrombus extension [55].

**Figure 2:**
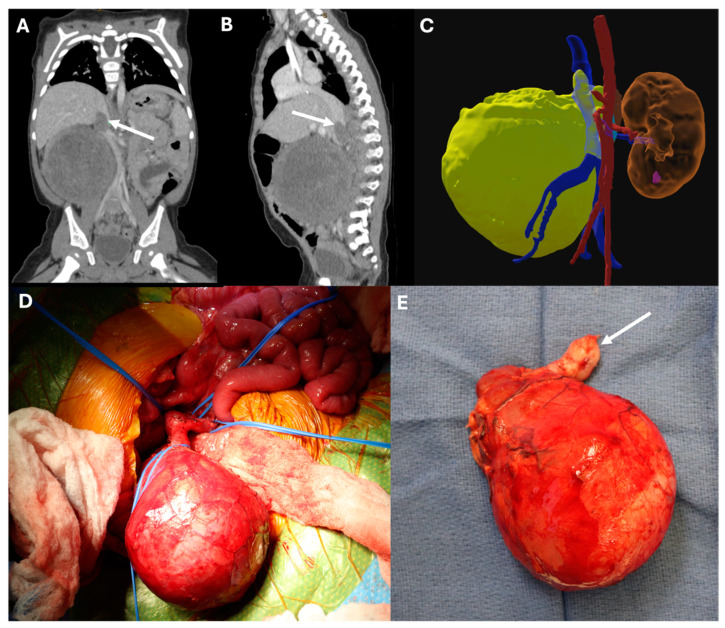
A five-year-old female presented with a right-sided Wilms tumor (WT) with infrahepatic inferior vena cava (IVC) extension. She underwent neoadjuvant chemotherapy followed by nephrectomy, cavotomy, and thrombectomy. (**A**) Preoperative computed tomography (CT) demonstrating a right-sided WT with infrahepatic IVC thrombus (arrow) in the coronal plane and (**B**) sagittal plane. (**C**) Three-dimensional reconstruction of preoperative CT, showing the right-sided WT and intravascular thrombus (yellow) within the IVC (blue) and iliac veins, with adjacent aorta (red) and left kidney (brown). (**D**) Intraoperative photo with the patient’s head towards the top left, demonstrating the right-sided mass and vascular isolation with vessel loops around the right renal vein (bottom), infrarenal IVC (right), left renal vein (top right), and suprarenal IVC (top left) prior to cavotomy and thrombectomy. (**E**) Final surgical specimen, demonstrating vena cava thrombus (arrow) removed en bloc with the right kidney and tumor.

Infrahepatic tumor thrombus (Daum class 1) generally does not require CPB, heparinization, or cardiac arrest for complete resection due to the ability to clamp the IVC above the level of the thrombus with minimal dissection and thus prevent distal embolism of the thrombus and back-bleeding from the cephalad IVC. These cases can be approached with a bisubcostal, transverse, or midline laparotomy. The tumor and adjacent kidney should be mobilized outside of Gerota’s fascia. The renal pedicle is dissected carefully to identify the ureter, renal artery, and renal vein with their respective insertions into the aorta and IVC. Adrenal veins should also be carefully dissected, and occlusion may be required during tumor thrombus resection to prevent back-bleeding. The artery and ureter are then ligated between ligatures. The ipsilateral renal vein is isolated with vessel loops or umbilical tape. The infrarenal IVC, contralateral renal vein, and suprarenal IVC are similarly isolated for vascular control, taking care to isolate the suprarenal IVC cephalad to the distal-most extent of tumor thrombus, as determined by manual palpation or intraoperative Doppler ultrasonography to avoid embolization. After controlling the contralateral renal vein and cephalad and caudad IVC, a longitudinal cavotomy is made, and the thrombus is retrieved en bloc with the kidney and tumor. Extension of the cavotomy to include elliptical excision of the involved renal vein orifice will facilitate en bloc resection of the tumor and, ultimately, caval repair. In some cases, the thrombus can be retrieved by bluntly dissecting it away from the vein intima using a freer elevator. The cavotomy can then be repaired primarily with polypropylene suture, closing the ipsilateral renal vein orifice contiguously (Figure 2; see the Supplementary Materials Video S1 for a video example).

In cases of adherence of the thrombus to the vessel wall with persistent flow around the thrombus, partial cavectomy may be required, followed by repair with a bovine pericardial patch to restore the appropriate caliber to the vessel [51,54]. However, complete IVC occlusion due to thrombus as seen on Doppler ultrasonography either pre- or intraoperatively typically signifies invasion of the thrombus into the vessel wall and therefore may warrant cavectomy of the affected segment and contralateral renal vein ligation. This is safe and usually well tolerated without reconstruction due to the prior development of venous collateral flow [28,55,58,59]. Cavectomy has the additional potential advantage of obviating the need for radiation therapy postoperatively for microscopic positive margins or incomplete resection [60].

In cases of tumor thrombus extension to the retrohepatic cava (Daum class 2), the right lobe of the liver will need to be mobilized to gain retrohepatic or suprahepatic IVC control. The retrohepatic IVC is dissected, and any small hepatic vessels directly draining to the IVC are carefully ligated [19]. This is essential to prevent back-bleeding when cavotomy is performed. The porta hepatis is dissected, and total hepatic vascular exclusion (Pringle maneuver) is necessary to avoid hepatic engorgement [20]. Cavotomy, partial cavectomy, or segmental cavectomy is then performed as above, with primary, patch, or graft closure as necessary. Hepatic vascular exclusion time should be limited, and once the thrombus is cleared from the retrohepatic cava, the cephalad IVC control can be moved to the infrahepatic cava, allowing the release of the porta hepatis blood flow. 

Extension of thrombus to the suprahepatic, extracardiac IVC (Daum class 3) should be approached through a midline laparotomy or chevron incision. Thrombus with subdiaphragmatic extension or even diaphragmatic extension may be able to be “milked” caudally to allow intraabdominal IVC control [19]. However, a mini-sternotomy for supradiaphragmatic, intrapericardial cava control may be necessary. Alternatively, the diaphragm can be divided to gain access to the supradiaphragmatic, intrapericardial IVC without necessitating a sternotomy [61]. For complete tumor thrombus resection, RA clamping may be necessary. In either case, hepatic vascular exclusion will be needed as described above, and CPB will likely be avoided.

**Figure 3:**
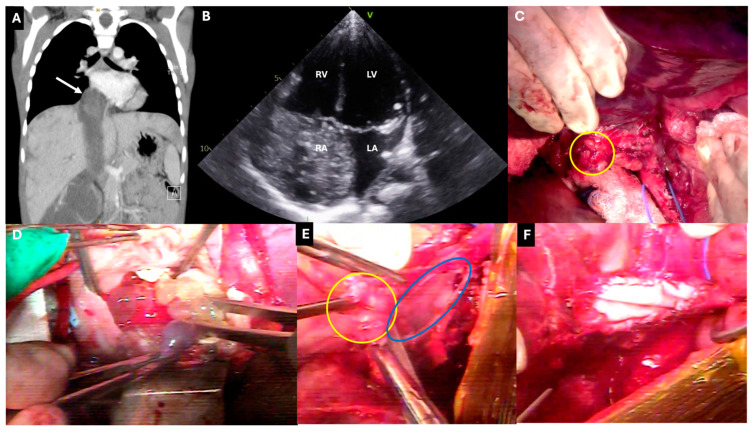
A 5-year-old female presented with a large right kidney mass with intravascular extension to the inferior vena cava (IVC) and right atrium (RA). She underwent neoadjuvant chemotherapy followed by local control surgery. Final pathology was reported as clear cell sarcoma of the kidney. (**A**) Preoperative computed tomography after neoadjuvant therapy demonstrating persistent right atrial thrombus (arrow) in the coronal plane. (**B**) Preoperative transthoracic echocardiogram with a heterogeneous, echogenic mass in the RA. (**C**) Intraoperative photograph after resection of right kidney and tumor, with the right renal vein obliterated by tumor and divided near its confluence with the IVC (yellow circle), a blue vessel loop around the infrarenal IVC (bottom), the liver reflected cephalad (top), and abdominal viscera reflected towards the patient’s left (right on photograph). (**D**) Delivery of a heterogeneous mass in forceps out of the right atrium after cardiopulmonary bypass initiation. (**E**) Piecemeal resection of tumor thrombus (yellow circle) during partial cavectomy (blue oval) of retrohepatic IVC, with the patient’s liver reflected cephalad (bottom left). (**F**) Following bovine pericardial patch repair of partial retrohepatic cavectomy (see the Supplementary Materials Video S2 for a video of intraoperative steps from this case).

CPB will be required to remove a tumor thrombus that extends to the RA and/or right ventricle (RV) (Daum class 4). These cases should be performed in conjunction with an experienced cardiothoracic surgery team (Figure 3; see the Supplementary Materials Video S2 for a video example). Either before or after mobilization of the kidney and tumor outside of Gerota’s fascia, a median sternotomy is performed, connecting to the abdominal chevron incision or midline laparotomy. The diaphragm is divided anteriorly, and the right lobe of the liver is mobilized to expose the entire IVC up to the RA. Control of the caudal IVC and the contralateral renal vein is obtained as above. The patient is heparinized and cannulated for CPB via the ascending aorta, the RA, and the superior vena cava [39,54]. Moderate-to-deep hypothermia is established with or without aortic cross-clamping and circulatory arrest. Thrombectomy is performed via cavotomy and possibly right atriotomy, depending on the extent of tumor thrombus infiltration into the cardiac chambers. Radical nephroureterectomy with en bloc thrombectomy is then completed. After repairing the IVC primarily or with a patch and closing the RA, hemostasis is achieved, the patient is rewarmed, and circulatory arrest and CPB are discontinued.

The advantages of performing tumor thrombectomy on CPB with deep hypothermia and circulatory arrest include operating in a near bloodless surgical field and a reduced risk of cellular spreading, pulmonary embolization, and massive hemorrhage [62]. Disadvantages of employing deep hypothermic circulatory arrest include an increased risk of postoperative coagulopathy and bleeding and a low risk of neurologic sequalae. Performing the operation without hypothermic circulatory arrest allows for a shorter bypass time and reduces the risk of warm hepatic and renal ischemia, hypoxic liver impairment, and acute tubular necrosis.

Other less common types of pediatric abdominal tumors such as clear cell sarcoma of the kidney (such as in Figure 3), neuroblastoma, adrenocortical carcinoma, renal primitive neuroectodermal tumor, and spindle cell sarcoma can exhibit intravascular extension beyond the renal vein [28,55,63,64]. From a technical perspective, their operative management is similar to what is described above. However, the principles of neoadjuvant and adjuvant therapy management and long-term outcomes in each entity are variable and are outside the scope of this review.

## 6. Outcomes

### 6.1. Surgical Complications

The presence of intravascular extension in patients with WT has been shown to significantly increase surgical morbidity, and the requirement of CPB for thrombectomy further increases morbidity, exposing the patient to the inherent risks of median sternotomy, atriotomy, blood product transfusion, systemic anticoagulation, and, in many cases, hypothermic cardiac arrest [8,62,65]. Nakayama et al. reported a complication rate of 11/15 (73%) among WT patients with cardiac thrombus in the first three NWTS studies who underwent primary surgery, with massive hemorrhage being the most common [10]. A surgical complication rate was reported at 43% for all patients with vena caval and/or cardiac involvement in the NWTS-3 study in which patients underwent primary nephrectomy. Massive hemorrhage was the most common complication, followed by distal embolism leading to cardiac arrest or hypotension from thrombus manipulation [24]. More contemporary multicenter studies have reported lower overall complication rates ranging from 15 to 30%. North American multicenter studies reported rates of 26% [8] and 30% [4], with significant improvement in bleeding and other early complication rates among patients requiring CPB in the more recent study by Naik-Mathuria et al. [4]. The SIOP 93-01/GPOH and SIOP 2001/GPOH studies had a complication rate of 18.2% among 33 patients, with 29/33 (87.9%) receiving neoadjuvant therapy [25]. The UKW3 study identified a similar complication rate of 9/59 (15.3%), with 8/59 patients (13.6%) experiencing massive hemorrhage intraoperatively, leading to 3 deaths [6]. A French multicenter study published in 2024 reported an overall complication rate of 12/53 (22.6%) when including both early and late complications [20]. Complications appear to occur at relatively high rates in cases where cavoatrial thrombus is not diagnosed preoperatively [10,24,25]. A collaborative approach between dedicated pediatric surgical oncology and pediatric cardiac surgery teams is imperative to help reduce surgical complications in cases where CPB is employed [4].

### 6.2. Long-Term Survival Outcomes

While intravascular extension complicates surgical management, long-term survival outcomes are favorable at 70–90%, which is comparable to patients without intravascular extension when complete thrombectomy is successfully performed [4,6,8,25]. Shamberger et al. demonstrated that, among children with WT and intravascular extension, there were no early perioperative deaths [8]. In this study, patients with stage IV disease at diagnosis had relatively poor relapse-free survival compared to those with stage II or III, and those with anaplastic histology suffered relatively poor EFS when compared to those with favorable histology; these trends were similar to patients without intravascular extension [8]. However, unadjusted and histology- and stage-adjusted 3-year EFS rates in this study were similar between patients with and without intravascular extension. Similarly, Meier and colleagues found lower OS among children in the SIOP/GPOH trials with intravascular extension when compared to those without (90.1% versus 97.8%, respectively, *p* < 0.001). Still, this association was confounded by higher proportions of patients with stage IV disease without complete remission and those with diffuse anaplastic histology in the group with intravascular extension [21]. Another contemporary study by Brener et al. created a multivariate logistic regression model for overall survival for children at their institution and found high-risk histology (*p* = 0.036), stage IV disease (*p* = 0.003), and sex (*p* = 0.046) to be significant but cavoatrial thrombus, capsule invasion or rupture, and local or metastatic site radiotherapy were not significant (*p* = 0.992, *p* = 0.374, *p* = 0.185, and *p* = 0.081, respectively) [13]. These data collectively serve to highlight that intravascular thrombus does not independently increase the odds of mortality when treatment is available and thrombectomy is successfully performed after accounting for other clinicopathologic factors. However, in poor-resource settings where access to multidisciplinary care is not readily available, higher rates of delayed presentation, progressive metastatic disease, and non-attempted or incomplete thrombectomy may be associated with inferior outcomes in patients with WT and intravascular extension [14,16,17,53,66,67]. Global disparities in WT care may furthermore lead to missing data from children whose tumors are deemed inoperable at diagnosis in these poor-resource settings [66,68,69].

As mentioned above, unfavorable histology and metastatic disease at presentation are clearly poor prognostic factors for patients with intravascular extension [4,7,8,13,28,70]. Though not certain, most data suggest that the thrombus level at diagnosis or post-neoadjuvant chemotherapy does not carry a prognostic value for long-term survival. One single-center study by Qureshi et al. found lower overall survival in patients with intracardiac extension compared to those with only IVC extension [71], but other groups have reported equivalent survival for these groups [5,20,25,27]. 

Other prognostic variables of interest include complete versus incomplete thrombectomy and the presence of viable tumor thrombus on the final pathology. Data for the effects of these factors is conflicting in many studies. A report of the IMPORT study by Dzhuma et al. noted a trend towards worse OS (log-rank *p* = 0.056) and significantly worse EFS (*p* = 0.00065) in incomplete thrombectomy, and tumor-related deaths following incomplete resection were mostly associated with viable thrombus [7]. However, this study found no differences in OS or EFS with viable thrombus on the final pathology. Other studies have noted relatively high rates of recurrence with incomplete thrombectomy: 1/6 (17%) with peritoneal relapse [31], 4/13 (31%) with pulmonary metastases [54], 2/18 with disease relapse [8], or 6/10 (60%) with fatal progressive disease [56]. Another large study by Naik-Mathuria found no association of survival with the completeness of thrombectomy or tumor thrombus viability [4]. Similar OS between groups in many studies suggests that adjuvant radiation therapy may at least partially overcome a negative influence on survival in cases of incomplete or non-attempted thrombectomy, which is classified as stage III disease [21]. Regarding these variable data, a commentary by Boam et al. advocated for an international trial to determine whether complete thrombectomy versus incomplete thrombectomy followed by adjuvant chemoradiotherapy is advantageous [72]. A meta-analysis of these above factors may also be informative to further quantify their significance in overall and relapse-free survival.

## 7. Conclusions

Wilms tumor with intravascular extension above the level of the renal veins is a rare manifestation occurring in 4–10% of cases that complicates surgical management. Most patients with intracaval tumor extension are diagnosed via imaging studies, and relatively few present with specific signs such as lower extremity swelling, dilated abdominal wall veins, Budd–Chiari syndrome, or cardiac failure. The degree of intravascular extension dictates the surgical approach, and hepatic vascular isolation and cardiopulmonary bypass increase the perioperative risks. Overall, the surgical complication rates have been reported at 15–30% in modern multicenter studies, with massive hemorrhage being most common. Neoadjuvant chemotherapy is indicated for tumor thrombus extension above the level of the hepatic veins and often leads to partial or complete thrombus regression after 4 to 6 weeks of therapy, thereby obviating the need for cardiopulmonary bypass in cases of cardiac thrombus at diagnosis. For patients with intravascular tumor extension only to the level of the retrohepatic cava, neoadjuvant therapy is not strictly indicated, though it may facilitate regression of the tumor thrombus and/or primary tumor, leading to a less-extensive surgical approach without hepatic vascular isolation. The rates of tumor thrombus complete or partial regression after neoadjuvant chemotherapy vary widely between recently published series, owing to the rarity of this presentation and the underlying biological heterogeneity of Wilms tumors. Following piecemeal, incomplete, or non-attempted thrombectomy, adjuvant chemoradiotherapy should be administered. With multimodal therapy, patients with vena caval and/or cardiac extension can achieve similar overall and event-free survival when compared to patients with WT without intravascular extension. However, patients with metastatic disease or unfavorable histology suffer relatively poor outcomes. Dedicated pediatric surgical oncology and pediatric cardiothoracic surgery teams, working together as part of a multidisciplinary team, are preferred for optimized outcomes in this patient population. 

## Figures and Tables

**Table 1:** Key studies assessing Wilms tumor with intravascular tumor extension with the response to neoadjuvant chemotherapy and surgical complication outcomes.

Study Author, Year	Study Type and Country/Countries	Study Total N	Thrombus Extent at Diagnosis	Stage at Diagnosis	Tumor Histology	Neoadjuvant Therapy Details	Response to Neoadjuvant Therapy	Surgical Approach	Surgical Complications
Ritchey 1993 [27]	Multicenter (United States)	30	IVC: 15 Cardiac: 15	III: 7 IV: 18 V: 5	Not provided	Received NC: 30/30 (100%) VA: 15/30 (50%) VAD: 14/30 (46.7%) Unknown regimen: 1/30 (3.3%) Received NRT: 7/30 (23.3%)	Thrombus: CR: 6/30 (20%) PR: 17/30 (56.7) NR: 5/30 (16.7%) Viable thrombus: 10/30 (33.3%)	Complete thrombectomy: 25/28 (89.3%); 7/25 with no IVC thrombus Incomplete thrombectomy: 3/28 (10.7%) Cardiac extension at diagnosis: 11/15 (73.3%) avoided sternotomy +/− CPB with NC	Overall: 7/28 (25%) Major hemorrhage > 50 cc/kg: 5/28 (17.9%) IVC occlusion: 2/28 (7.1%) Small bowel obstruction: 1/28 (3.6%) Perioperative death: 1/28 (3.6%); related to sternal dehiscence and sepsis
Mushtaq 1996 [29]	Multicenter (United Kingdom)	30	IVC: 16 (13 IH 13, 2 RH, 1 SH) Cardiac: 5 Unknown: 8	II: 17 III: 3 IV: 10	FH: 23/30 (76.7%) UH: 6/30 (20%) Clear cell sarcoma: 1/30 (3.3%)	Received NC: 21 Complications during NC: Tumor rupture: 3/21 (14.3%); 1 death from hemorrhage	Thrombus: CR/PR: 16/21 (76.2%) NR: 5/21 (23.8%)	Primary surgery: 9/30 (30%) Cavotomy: 8/21 (38.1%) with NC vs. 5/9 (55.6%) with primary surgery Incomplete thrombectomy: 2/21 (9.5%) with NC vs. 3/9 (33.3%) with primary surgery CPB: 2/21 (9.5%) with NC Cardiac extension at diagnosis: 2/3 (66.7%) avoided CPB with NC	Not provided
Shamberger 2001 [8]	Multicenter (multinational) NWTS-4	165	IVC: 134 Cardiac: 31	Not provided	FH: 140/152 (92.1%) FA: 2/152 (1.3%) DA: 7/152 (4.6%) Clear cell sarcoma 3/152 (2%)	Received NC: 69/165 (41.8%); 55 IVC, 13 cardiac VA: 22 VAD: 45 VAD + cyclophosphamide: 2 Received NRT: 5 Complications during NC: 1 tumor embolism 3 acute respiratory distress syndrome (1 fatal) 1 progressive disease	Thrombus: PR/CR: 39/49 (80%) 7/12 (58%) regressed from atrial to lower level Viable thrombus: 22/42 (52%) in NC vs. 75/75 (100%) in primary surgery	Thrombectomy not attempted: 18/164 (11.0%); 9/18 received NC IH IVC occlusion: 82/164 (50.0%) SH IVC occlusion: 21/164 (12.8%) Adherence to vessel wall: 61.7% with NC vs. 43.7% in primary surgery, *p* = 0.04 Partial cavectomy: 8/164 (4.9%) Total cavectomy and reconstruction: 2/164 (1.2%) CPB: 9/69 (13.0%) with NC vs. 19/96 (19.8%) with primary surgery Cardiac extension at diagnosis: 7/12 (58%) avoided CPB with NC	By thrombus extent: 23/134 (17.2%) in IVC thrombus, 11/30 (36.7%) atrial, *p* = 0.025 By NC: 13/68 (19%) with NC when including complications during NC vs. 25/96 (26%) with primary surgery, *p* = 0.35 Local spillage: 65 patients with IVC, 17 with atrial thrombus Diffuse spillage: 12 patients with IVC, 7 with atrial thrombus
Szavay 2004 [25]	Multicenter (multinational) SIOP 93-01/GPOH and SIOP 2001/GPOH	33	IVC: 24 Cardiac: 9	IV: 16 V: 1	Not provided	Received NC: 29/33 (87.9%) VA: 17, 4 weeks VAD: 16 (with stage IV disease)	Primary tumor: >75% PR/CR: 9/29 (31.0%) 50–75% PR: 4/29 (13.8%) 25–50% PR: 6/29 (20.7%) <25% or NR: 7/29 (24.1%) Unknown: 3/24 (10.3%)	Primary surgery: 4/33 (12.1%) CPB: 9/33 (27.3%) Complete resection: 27/33 (81.8%) Incomplete resection: 5/33 (15.2%); 2 with IVC, 3 with atrial thrombus	Overall: 6/33 (18.2%) Tumor spillage: 3/28 (10.7%) Perioperative death: 0/33 (0%) Late: IVC occlusion: 2/33 (6.1%)
Akyüz 2005 [56]	Single center (Turkey)	17	IVC: 15 Cardiac: 2	III: 7 IV: 9 V: 1	FH: 14/17 (82.4%) UH: 3/17 (17.6%)	Received NC: 14/17 (82.4%) VA: 14/14 (100%), median 4 weeks, range 1–12 weeks	Thrombus: CR: 2/14 (14.3%); 1 IH, 1 cardiac thrombus PR: 8/14 (57.1%) NR or unknown: 4/14 (27.6%) Viable thrombus: 0/2 (0%) Primary tumor: PR >50%: 10/17 (58.8%)	No IVC thrombus found: 1/17 (5.9%) Thrombectomy attempted after NC: 2/14 (14.3%); 2/2 incomplete Thrombectomy not attempted after NC: 12/14 (87.5%)	Not provided
Lall 2006 [6]	Multicenter (United Kingdom, Ireland) UKW3	59	IVC: 49 (26 IH, 8 RH, 9 SH, 6 unknown) Cardiac: 10	Not provided	FH: 56/59 (94.9%) UH: 3/59 (5.1%)	Received NC: 52/59 (88.1%) VA: 7 VAD: 45, 36/45 for 9+ weeks Complications during NC: Tumor rupture: 3/52 (5.8%)	Thrombus: PR/CR: 35/49 (71.4%) NR: 8/49 (16.3%)	Approach: Primary surgery: 6/59 (10.2%) Cavotomy/cavectomy: 31/59 (52.5%) Cavectomy: 11/59 (18.6%); all underwent end-to-end anastomosis Cardiac extension at diagnosis: 7/10 (70%) avoided CPB with NC Intraoperative findings: No IVC thrombus found: 21/59 (35.6%) Fibrotic/calcified IVC: 8/52 (15.4%) with NC	Significant hemorrhage: 8/59 (13.6%); controlled in 5, lead to death in 3–all with poor response to NC Pulmonary embolism: 1/59 (1.7%) Death: 3/59 (5.1%)
Cristofani 2007 [15]	Single center (Brazil)	16	IVC: 8 (5 IH, 3 SH) Cardiac: 8	II: 6 III: 7 IV: 3	FH: 13/16 (81.3%) UH: 3/16 (18.7%)	Received NC: 11/16 (68.8%) VA: 11/11 (100%), 7 IVC, 4 cardiac thrombus; 4–6 weeks Received NRT: 0/16 (0%)	Thrombus: CR: 2/11 (18.2%) PR: 6/11 (54.5%) NR: 3/11 (27.3%) Viable thrombus: 6/11 (54.5%), all FH tumors	Primary surgery: 5/16 (31.3%) CPB: 6/11 (54.5%) Atrial extension at diagnosis: 2/4 (50%) avoided CPB with NC	Infection: 2/11 (18.2%); 1 with NC, 1 with primary surgery
Hadley 2010 [17]	Single center (South Africa)	40	IVC: 30 (16 IH, 14 RH) Cardiac: 10	II/III: 17 IV: 17 V: 2 Unknown: 4	FH: 24/27 (88.9%) UH: 3/27 (11.1%); 2/3 with FA, 1/3 blastemal	Received NC: 40/40 (100%) Complications during NC: Neutropenic sepsis: 3/40 (7.5%); 3/3 led to death Death: 5/40 (12.5%)	Thrombus: CR: 0/40 (0%) PR: 18/40 (45%) NR/progressive: 22/40 (55%) Viable thrombus: 24/31 (77.4%)	Underwent surgery: 31/40 (77.5%); 5 died preop, 4 refused surgery Laparotomy only: 24/31 (77.4%) Cavotomy: 23/31 (74.2%) Cavectomy: 1/31 (3.2%) Cardiac extension at diagnosis: 3/10 (30%) avoided CPB with NC CPB: 7/31(22.6%)	Not provided
Abdullah 2013 [11]	Single center (South Africa)	9	Cardiac: 9 (7 RA, 2 RV)	III: 4 IV: 5	FH: 9/9 (100%)	Received NC: 9/9 (100%) VAD: 6/9 (66.7%) Complications during NC: Death: 1/9 (11.1%); due to septicemia after 2 doses NC	Thrombus: CR: 0/9 (0%) PR: 3/9 (33.3%) NR: 5/9 (55.6%) Unknown: 1/9 (11.1%)	Emergent surgery: 1/8 (12.5%); due to tricuspid valve occlusion CPB: 6/8 (75.0%) Cardiac extension at diagnosis: 2/8 (25.0%) avoided CPB with NC Abdominal cavotomy: 5/8 (52.5%) Cavectomy: 3/8 (37.5%)	Death: 1/8 (12.5%); from massive hemorrhage during delayed thrombectomy on CPB Emergent CPB: 1/8 (12.5%)
Aspiazu 2012 [57]	Single center (Spain)	7	IVC: 1 (1 IH) Cardiac: 6	IV: 2	FH: 6/7 (85.7%) UH: 1/7 (14.3%)	Received NC: 7/7 (100%) VAD: 7/7 (100%)	Thrombus: CR: 1/7 (14.7%) PR: 3/7 (42.9%) NR: 3/7 (42.9%); all Daum IV	No IVC thrombus found: 1/7 (14.3%) Cavotomy 2/7 (28.6%) CPB: 4/7 (57.1%) Cardiac extension at diagnosis: 2/6 (33.3%) avoided CPB with NC	Postoperative hemorrhage: 2/7 (28.6%) Recurrent IVC thrombus: 1/7 (14.3%)
Loh 2015 [28]	Single center (United States)	12	IVC: 9 (IH 6, 1 RH, 1 SH, 1 unknown) Cardiac: 3	II: 1 III: 4 IV: 6 V: 1	FH: 9/12 (75%) UH: 3/12 (25%)	Received NC: 10/12 (83.3%)	Thrombus: NR: 4/4 (100%) of Hinman III; not provided for other groups Viable thrombus: 7/12 (58.3%)	Primary surgery: 2/12 (16.7%) Thrombectomy not attempted: 1/12 (8.3%) Complete thrombectomy: 4/12 (33.3%) Incomplete thrombectomy: 6/12 (50%) CPB: 1/12 (8.3%) Partial cavectomy and patch reconstruction: 1/12 (8.3%)	Overall: 2/10 (20%) with NC Intraoperative CPR: 1/12 (8.3%)
Al Diab 2017 [26]	Single center (Jordan)	11	IVC: 6 Cardiac: 5	IV: 5 V: 5	FH: 10/11 (90.9%) UH: 1/11 (9.1%)	Received NC: 10/11 (90.9%), median 7 weeks (range 0–12)	Thrombus: CR: 4/10 (40%), all Hinman I PR: 5/10 (50%), all Hinman III NR: 1/10 (10%) (Hinman I) Primary tumor: Tumor diameter: Median 11 cm (range 1.4–21) pre-NC vs. 8.9 cm (range 1.3–15), 19% diameter reduction Pulmonary metastases: CR: 1/5 (20%) PR: 3/5 (60%) Stable disease: 1/5 (20%)	Complete thrombectomy: 11/11 (100%) Laparotomy: 11/11 (100%) Adherence to vessel wall: 7/11 (63.6%) Cavectomy and reconstruction: 1/11 (9.1%) Cardiac extension at diagnosis: 5/5 (100%) avoided CPB with NC CPB: 0/11 (0%); all cardiac extension at diagnosis with sufficient thrombus response	Local tumor spillage: 1/11 (9.1%)
Cox 2018 [14]	Single center (South Africa)	12	Cardiac: 12	III: 8 IV: 4	FH: 11/11 (100%)	Received NC: 12/12 (100%) VAD: 12/12 (100%); for <8 weeks in 3/12,12 weeks in 5/12, 26 weeks in 3/12 Complications during NC: Death: 1/12 (8.3%); due to sepsis and respiratory arrest	Thrombus: PR: 2/11 (18.2%) NR: 9/11 (81.8%) Viable thrombus: 9/11 (81.8%)	Emergency surgery: 1/11 (9.1%) Laparotomy only: 2/11 (18.2%) CPB: 9/11 (81.8%) Cardiac extension at diagnosis: 2/11 (18.2%) avoided CPB with NC Cavotomy: 7/11 (63.6%) Cavectomy: 4/11 (36.4%) Partial atrial resection: 2/11 (18.2%) Cavoatrial patch: 5/11 (45.5%); 3/5 autologous pericardial, 2/5 PTFE	Emergent CPB: 1/11 (9.1%) Death: 1/11 (9.1%); due to massive hemorrhage
Xu 2019 [31]	Single center (China)	42	Renal vein: 5 IVC: 27 (21 RH, 6 SH) Cardiac: 10	II: 20 III: 9 IV: 13	LR: 14/42 (33.3%); all necrotic Mixed: 18/42 (42.9%) Mesenchymal: 5/42 (11.9%) Germ: 3/42 (7.1%) germ Anaplastic: 1/42 (2.3%)	Received NC: 36/42 (87.1%) Received NRT: 3/42 (7.1%)	Thrombus: CR/PR: 26/36 (72.2%) Primary tumor: CR/PR: 31/36 (86.1%)	Primary surgery: 6/42 (14.3%) En bloc thrombectomy: 36/42 (85.7%) Piecemeal thrombectomy: 6/42 (14.3%) Cardiac extension at diagnosis: 5/10 (50%) avoided CPB with NC CPB: 5/42 (11.9%)	Death: 0/42 (0%)
Elayadi 2020 [5]	Single center (Egypt)	Total N: 51	IVC: 48 (33 IH, 9 RH, 6 SH) Cardiac: 3	III: 22 IV: 25 V: 4	FH: 47/51 (92.2%) UH: 4/51 (7.8%)	Received NC: 50/51 (98%) VAD: 50/50 (100%); 6–12 weeks	Thrombus: CR: 16/50 (32.0%); all IH, PR: 24/50 (48.0%); 16 IH, 2 RH, 4 SH, 2 cardiac NR: 8/50 (16.0%); 6 RH, 1 SH, 1 cardiac Progressive: 1/50 (2.0%); RH at diagnosis Unknown: 1/50 (2.0%); SH at diagnosis Viable thrombus: 20/31 (64.5%) Length of thrombus: Mean 6.5 cm (range 1.5–22.5) pre-NC vs. 3.6 cm (range 0–16) post-NC Primary tumor: Tumor volume: Median 782 cm^3^ pre-NC vs. 167.6 cm^3^ post-NC; 79% volume reduction	Primary surgery: 1/51 (2.0%) Complete thrombectomy: 50/51 (98.0%) Thrombectomy not attempted: 1/51 (2.0%); cardiac thrombus after NC and received adjuvant RT CPB: 0/51 (0%); not available at this center	Overall: 0/51 (0%)
Qureshi 2021 [30]	Single center (India)	43	Renal vein: 5 IVC: 26 (12 IH, 14 RH) Cardiac: 11 Unknown: 1	II/III: 25 IV: 17 V: 1	IR: 38/42 (90.5%) HR: 4/42 (9.5%)	Received NC: 42/43 (97.7%) VA: 12/42 (28.6%); median 6 weeks (range 4–7) VAD: 30/42 (71.4%); median 8 weeks (range 4–12)	Thrombus radiologic response: CR: 0/41 (0%) PR: 11/41 (26.8%) NR: 30/41 (73.2%) Thrombus clinical response: CR: 6/42 (14.3%) PR: 4/42 (9.5%) Viable thrombus: 26/36 (72.2%)	Emergency surgery: 1/43 (2.3%); due to tumor rupture pre-NC No IVC thrombus found: 6/43 (14%) Complete thrombectomy: 35/43 (81.4%); piecemeal 10/37 Incomplete thrombectomy 2/43 (4.7%) Cardiac extension at diagnosis: 4/11 (36.4%) avoided CPB with NC CPB: 7/11 (63.6%)	Massive hemorrhage >50 mL/kg: 3/43 (7%); 1/3 led to death Recurrent IVC thrombus: 1/43 (2.3%); received anticoagulation Obturator nerve injury: 1/43 (2.3%) Bowel obstruction: 1/43 (2.3%); required adhesiolysis Death: 1/43 (2.3%); following re-operation for hemorrhage
Dzhuma 2022 [7]	Multicenter (United Kingdom, Ireland) IMPORT	69	Renal vein: 14 IVC: 37 (21 IH, 10 RH, 6 SH) Cardiac: 8 Unknown: 10	Unilateral: 60 Bilateral: 9 Localized tumor: 40 Metastatic: 29	LR: 8/69 (11.6%) IR: 54/69 (78.3%) HR: 7/69 (10.1%)	Received NC: 68/69 (98.6%) VA: 38/68 (55.9%) VAD: 29/68 (42.6%) Other: 1/68 (1.5%)	Thrombus: CR/PR: 13/59 (22%) NR: 44/59 (74.6%) Progressive: 2/59 (3.4%) Viable thrombus: 45/68 (66.2%)	Thrombectomy not attempted: 3/69 (4.3%) Complete thrombectomy: 58/69 (84.1%); 20/58 piecemeal Incomplete thrombectomy: 8/69 (13.6%) CPB: 15/69 (21.7%) Partial cavectomy with patch repair: 11/69 (15.9%)	Not provided
Fanelli 2022 [55]	Single center (Brazil)	34	Not provided	Not provided	Tumor types: 21 WT 11 adrenocortical carcinoma 1 renal primitive neuroectodermal tumor 1 hepatoblastoma	Received NC: 21/21 (100%)	Thrombus: CR: 5/21 (23.8%)	Not provided for WT sub-group	Not provided
Meier 2022 [21]	Multicenter (Austria, Switzerland, Germany) SIOP-9/GPOH, SIOP-93-01/GPOH and SIOP-2001/GPOH	148	IVC: 95 (78 IH, 5 RH, 12 SH) Cardiac: 20 Unknown: 30	Metastatic disease: 81 Bilateral: 8	LR: 17/148 (11.5%) IR: 119/148 (80.4%) HR: 12/148 (8.1%)	Received NC: 142/148 (95.9%)	Thrombus: CR: 14/113 (12.4%) PR: 12/113 (10.6%) NR: 86/113 (76.1%) Progressive: 1/113 (0.9%); UH, SH at diagnosis	Primary surgery: 5/148 (3.4%) No IVC thrombus found: 14/148 (9.5%) Complete thrombectomy: 111/130 (85.4%) Incomplete thrombectomy: 16/130 (12.3%) Thrombectomy not attempted: 3/130 (2.3%); all IH Cardiac extension at diagnosis: 6/20 (30%) avoided CPB with NC CPB: 10/130 (7.7%) Cavectomy with prosthetic graft: 13/130 (10%)	Deaths: 0/148 (0%)
Naik-Mathuria 2024 [4]	Multicenter (United States)	124	IVC: 99 (53 IH, 32 RH, 14 SH) Cardiac: 24	II: 4 III: 44 IV: 63 V: 12	FH: 81% UH: 12% Other/unknown: 9%	Received NC: 102/124 (82.3%) VA: 4/102 (3.9%) VAD: 82/102 (80.4%) Other regimen: 16/102 (15.7%) Complications during NC: Massive hemorrhage: 1/102 (1%) Fungal infection: 1/102 (1%) Pulmonary embolism: 1/102 (1%)	Thrombus: CR: 19/95 (20%) PR: 24/95 (25.3%) NR: 50/95 (52.6%) Progressive: 2/95 (2.1%) Viable thrombus: 36/99 (36%)	Approach: Primary surgery: 19/124 (15.3%) CPB: 14/124 (11.3%); 12/14 with NC Following NC (data not provided for primary surgery): No IVC thrombus found: 19/102 (18.6%) Thrombectomy not attempted: 3/102 (2.4%); all Daum IV Cardiac extension at diagnosis: 10/22 (45.5%) avoided CPB Cavectomy without reconstruction: 2/102 (2%) Cavotomy with patch repair: 4/102 (3.9%)	Intraoperative/Early: Overall: 37/124 (29.9%); 25/102 (24.5%) with NC vs. 12/22 (54.5%) with early surgery, *p* = 0.005 Death: 0/124 (0%) Massive hemorrhage: 7/124 (5.6%); 4/102 (3.9%) with NC vs. 3/22 (13.6%) with early surgery Tumor rupture: 5/124 (4%); 2/102 (2%) with NC vs. 3/22 (13.6%) with early surgery Bowel obstruction: 4/124 (3.2%); 3/102 (2.9%) with NC vs. 1/22 (4.5%) with early surgery Acute kidney injury: 3/124 (2.4%); 1/102 (1%) with NC vs. 2/22 (9.1%) with early surgery Infection: 8/124 (6.5%); 8/102 (7.8%) with NC vs. 0/22 (0%) with early surgery Symptomatic pericardial effusion: 1/124 (0.8%); received NC Repeat operation: 4/124 (3.2%); 3/102 (2.9%) with NC vs. 1/22 (4.5%) with early surgery Pulmonary embolus from recurrent thrombus:1/124 (0.8%); received NC Other: 4/124 (3.2%); 3/102 (2.9%) with NC vs. 1/22 (4.5%) with early surgery Late: IVC stenosis: 13/124 (10.5%) Recurrent IVC thrombus: 13/124 (10.5%)
Pio 2024 [20]	Multicenter (France)	69	IVC: 40 (29 IH, 9 RH, SH 2) Cardiac: 24 Unknown: 5	II: 8 III: IV 13 V: 2	IR: 69/69 (100%); 4/69 with focal anaplasia	Received NC: 67/69 (97.1%)	Thrombus: PR/CR: 21/59 (35.6%) NR: 38/59 (64.4%) Viable thrombus: 22/43 (51.2%)	Cavotomy: 47/62 (75.8%) Cavectomy 13/62 (20.9%); 8/13 Gore-Tex tube, 1/13 pericardial patch, 1/13 primary repair, 1/13 without reconstruction Cardiac extension at diagnosis: 10/24 (41.7%) avoided CPB with NC En bloc thrombectomy: 42/60 (70%) Piecemeal thrombectomy: 18/60 (30%)	Overall: 12/53 (22.6%) Intraoperative/early: 4/53 (7.5%) Massive hemorrhage: 3/53 (5.7%); 1/3 led to death Bowel obstruction: 1/52 (1.9%); required laparotomy Death: 1/53 (1.9%) Late: 8/53 (15.1%) Recurrent IVC thrombus: 5/52 (9.4%) Acute renal failure: 3/53 (5.7%); 3/3 led to death Death: 3/53 (5.7%)

WT, Wilms tumor; IVC, inferior vena cava; IH, infrahepatic; RH, retrohepatic; SH, suprahepatic; FH, favorable histology; UH, unfavorable histology; FA, focal anaplasia; DA, diffuse anaplasia; LR, low risk; IR, intermediate risk; HR, high risk; NC, neoadjuvant chemotherapy; NRT, neoadjuvant radiation therapy; VA, vincristine/actinomycin D; VAD, vincristine/actinomycin D/doxorubicin; CR, complete response; PR, partial response; NR, no response; CPB, cardiopulmonary bypass.

## Data Availability

No new data were generated or collected regarding this research.

## Supplementary Materials

The following supporting information can be downloaded at https://doi.org/10.5281/zenodo.12610924 (Video S1: Right nephrectomy with IVC thrombectomy) and https://doi.org/10.5281/zenodo.12611089 (Video S2: Right nephrectomy with RA thrombectomy).
